# Statistical optimization of acid phosphatase production by *Trichoderma* spp. in submerged fermentation

**DOI:** 10.1007/s13205-026-04817-9

**Published:** 2026-05-02

**Authors:** Frederico Alves Lima, Jussara Maria Martins de Almeida Afonso, Amanda Rocha Carmelo, Miriam Maria de Resende

**Affiliations:** Chemical Engineering Faculty, Federal University of Uberlândia, Av. João Naves de Ávila 2121, Campus Santa Mônica, Bloco 1K, P.O. Box 593, Uberlândia, MG 38408-144 Brazil

**Keywords:** Acid phosphatase, *Trichoderma spp*., *T. asperellum*, *T. harzianum*, *T. reesei*, Culture medium optimization, SDS-PAGE

## Abstract

The production of acid phosphatase (ACPase) by *Trichoderma spp*. was evaluated and optimized under submerged fermentation using low-cost culture media. Among the strains tested, *T. reesei* showed the highest enzymatic potential. A Central Composite Design was applied to assess the effects of sucrose, yeast extract, and KH₂PO₄ on ACPase activity. The optimized medium (MSO-Tr) resulted in a maximum enzymatic activity of 1.96 ± 0.14 U/mL and biomass production of 5.32 ± 0.48 g/L, representing a 3.3-fold increase compared to preliminary conditions. *T. harzianum* and *T. asperellum* showed lower activities, reaching 0.81 ± 0.05 U/mL and 0.76 ± 0.12 U/mL, respectively. Substrate consumption analysis indicated complete utilization of sucrose and glucose within 48 h for *T. reesei*. Response surface analysis demonstrated that yeast extract positively influenced enzyme production, whereas excess phosphate had inhibitory effects depending on the strain. SDS-PAGE analysis revealed protein bands in the 58–69 kDa range, consistent with the reported molecular weights of fungal acid phosphatases. These results demonstrate the effectiveness of statistical optimization and highlight the biotechnological potential of *Trichoderma*-derived ACPases produced using low-cost substrates.

## Introduction

Phosphorus (P) plays a fundamental role in agricultural production and is essential for plant growth; however, its availability in soils is often limited due to complex chemical interactions that reduce its assimilation by plants (Kumar et al. 2025). The bioavailability of P largely depends on the mineralization of organic forms through enzymatic processes mediated by phosphatases, which catalyze the hydrolytic cleavage of phosphate esters, releasing inorganic phosphate into the soil solution.

The mineralization of rock phosphate (RP) and other insoluble phosphorus sources is driven by microbial activity, including the secretion of organic acids, proton extrusion, and the production of phosphatases. These enzymes play a crucial role in soil phosphorus cycling and are influenced by environmental factors such as pH, temperature, nutrient availability, and microbial biomass (Margalef et al. 2021). Therefore, phosphatase activity is widely used as an indicator of soil quality and nutrient dynamics.

Fungi are key contributors to the decomposition of organic matter and the cycling of nutrients. They are highly efficient at converting agricultural residues. *Trichoderma spp.*, in particular, are widely recognized for their ecological versatility and multifunctional roles, including enzyme production, promoting plant growth, and exhibiting biocontrol activity (Chen et al. 2025). They can enhance nutrient availability and plant development through various biological mechanisms, such as the secretion of extracellular enzymes relevant to biotechnology (Prismantoro et al. 2025).

Among the species of this genus, *Trichoderma reesei* has been extensively studied due to its remarkable capacity for enzyme secretion, particularly those involved in biomass degradation, making it an important model organism in biotechnology (Zhao et al. 2023). Similarly, *Trichoderma harzianum* is widely recognized for its role in plant protection and enzyme production, while *Trichoderma asperellum* has been associated with plant growth promotion and the induction of systemic resistance through the secretion of hydrolytic enzymes (Guo et al. 2021).

In addition to their roles in plant–soil interactions, *Trichoderma* species contribute to phosphorus mobilization through enzymatic and metabolic mechanisms, reinforcing their importance in sustainable agriculture. Microbial enzymes are valuable biocatalysts with broad industrial applications, and *Trichoderma spp.* stand out due to their ability to produce a wide range of enzymes under different cultivation conditions (Wang et al. 2024).

In recent years, increasing attention has been given to the use of filamentous fungi for sustainable enzyme production, particularly through process optimization strategies and the use of low-cost substrates (Greco-Duarte et al. 2023; Wang et al. 2024). Despite these advances, studies focusing on the optimization of acid phosphatase production by *Trichoderma spp.* under submerged fermentation conditions remain limited, especially when considering integrated approaches combining medium composition, nutrient sources, and statistical optimization tools.

Submerged fermentation (SmF) is a well-established and controllable process widely used to produce microbial enzymes, offering advantages such as process homogeneity and ease of parameter control compared to solid-state fermentation. However, the efficiency of enzyme production under SmF strongly depends on medium composition and cultivation conditions, requiring systematic optimization.

Therefore, this study aimed to evaluate and statistically optimize acid phosphatase production by *Trichoderma harzianum*, *Trichoderma reesei*, and *Trichoderma asperellum* under submerged fermentation using low-cost culture media. The findings provide insights into process optimization and highlight the biotechnological potential of these microorganisms for sustainable enzyme production.

## Materials and methods

### Preservation and activation of microorganisms

The fungal strains (*Trichoderma asperellum*, *Trichoderma harzianum*, and *Trichoderma reesei*) were previously isolated from the Araxá Mining Complex (Vale Fertilizantes, Minas Gerais, Brazil), identified by the André Tosello Research Foundation (Campinas, SP, Brazil), and preserved at − 70 °C, as described by Lima et al. (2022). After preservation, the strains were reactivated by transferring preserved paper discs containing fungal propagules to Petri dishes containing Czapek agar medium, followed by incubation at 25 °C for 5–7 days until visible mycelial growth and sporulation were observed.

### Scanning electron microscopy (SEM)

Scanning electron microscopy (SEM) was used to evaluate the morphological characteristics of *Trichoderma spp.* qualitatively during submerged fermentation. The goal of this analysis was to provide additional information on fungal growth patterns under liquid culture conditions and support discussions on biomass development without establishing a direct quantitative correlation with enzyme activity. Sample preparation followed the Karnovsky fixation protocol previously described by de Almeida Afonso et al. (2025), with minor modifications. Briefly, fungal biomass samples were fixed in Karnovsky solution, dehydrated through a graded ethanol series, subjected to critical point drying, and sputter-coated with gold prior to analysis. SEM analyses were performed using a Zeiss EVO MA 10 microscope. The accelerating voltage (EHT) was adjusted according to the sample and imaging conditions, as reported in the corresponding figure captions. Images were acquired at different magnifications to evaluate hyphal organization and surface morphology.

### Determination of dry biomass and pH

Dry biomass concentration (g/L) was determined using a standard gravimetric method. Briefly, 15 mL culture samples were centrifuged at 8000 rpm (≈ 12,096 × g) for 10 min in a Beckman J-25 centrifuge. The supernatant was discarded, and the resulting pellet was washed with distilled water and transferred to a pre-weighed container. Samples were dried in an oven at 80 ± 1 °C until constant weight, and biomass concentration was calculated from the mass difference. The pH of the culture broth was measured using a calibrated Gehaka bench pH meter.

### Determination of substrate consumption by HPLC

Substrate consumption during submerged fermentation was analyzed by high-performance liquid chromatography (HPLC) by monitoring sucrose and glucose concentrations in the culture supernatant. Samples were collected at defined fermentation times and centrifuged to remove fungal biomass, and the resulting supernatants were used for chromatographic analysis. HPLC analyses were performed using a Shimadzu LC-20 A Prominence system equipped with a refractive index detector (RID) and a Supelcogel C-610 H column (300 × 7.8 mm). The mobile phase consisted of 0.1% (v/v) phosphoric acid, with a flow rate of 0.5 mL·min⁻¹, column temperature of 32 °C, and injection volume of 20 µL. Substrate concentrations were determined using external calibration curves obtained by linear regression. The calibration equation for sucrose was C = 3.53 × 10⁻⁶A + 7.21 × 10⁻³ (R² = 0.9997), while for glucose it was C = 3.06 × 10⁻⁶A + 3.41 × 10⁻² (R² = 0.9995), where C represents the sugar concentration and A the chromatographic peak area. Substrate identification was performed by comparison with standard retention times. Sucrose and glucose retention times were 11.0 and 14 0.0 min, respectively.

### Acid phosphatase activity assay

Acid phosphatase activity was determined using p-nitrophenyl phosphate (pNPP) as substrate, according to Leitão et al. (2010), with minor modifications. The reaction mixture consisted of 50 µL of enzyme extract, 100 µL of pNPP solution (5 mmol·L⁻¹), and 350 µL of sodium acetate buffer (50 mmol·L⁻¹, pH 5.0). The enzymatic reaction was carried out at 45 °C for 15 min in a thermostatic water bath and was stopped by the addition of 1000 µL of 0.1 mol·L⁻¹ NaOH. The amount of p-nitrophenol released was determined spectrophotometrically at 405 nm. One unit (1 U) of acid phosphatase activity was defined as the amount of enzyme required to release 1 µmol of p-nitrophenol per minute under the assay conditions, according to Ames (1966).

### Submerged fermentation in a shaker incubator

Submerged fermentations were carried out in an orbital shaking incubator. *T. asperellum*, *T. reesei*, and *T. harzianum* were inoculated separately into 500 mL Erlenmeyer flasks containing 200 mL of culture medium. The cultures were maintained at 25 °C under agitation at 150 rpm. The culture media used for acid phosphatase production were selected from the literature, and their compositions are presented in Table 1. During fermentation, cell biomass growth (g/L), acid phosphatase activity (U/mL), and pH were evaluated for each fungal strain. Fermentations were initiated using spore suspensions obtained from previously activated *Trichoderma spp.* cultures grown on liquid medium. The initial inoculum size was defined according to each fermentative process and added to the culture medium at the beginning of fermentation.

**Table 1:** Culture media used in submerged fermentation

Culture Medium	Reagents – Concentration (g/L)	References
Medium 1 – JP	Sucrose – 20.0 Yeast extract – 5.0	Farroupilha Laboratory
Medium 2 – Modified JP	Sucrose – 20.0 Yeast extract – 5.0 KH_2_PO_4_ – 2.0	Farroupilha Laboratory /UFU
Medium 3 – Liquid Czapek	Sucrose – 30.0 NaNO_3_ – 2.0 K_2_HPO_4_ – 1.0 MgSO_4_ – 0.5 KCl – 0.5 FeSO_4_ – 0.01	Adapted from Araújo (2014)
Medium 4 – Sampaio	Glucose – 10.0 Yeast extract – 0.5 MgSO_4_ – 0.1 MnSO_4_ – 0.001 FeSO_4_ – 0.001 (NH_4_)_2_SO_4_ – 0.5	Sampaio et al. (2003)
Medium 5 – Leitão	Glucose – 2.5 Bacterial peptone – 1.0 Urea – 0.3 KH_2_PO_4_ – 2.0 (NH_4_)_2_SO_4_ – 14.0 MgSO₄·7 H₂O – 0.3	Leitão et al. (2010)
Medium 6 – Souza	Glucose – 15.0 Yeast extract – 2.5 CaCl_2·_6H_2_O – 0.3 (NH_4_)_2_SO_4_ – 1.4 MgSO₄·7 H₂O – 0.3	Souza et al. (2016)

### Culture media

The culture media used in the fermentative process to produce acid phosphatases were selected from the literature. Table 1 shows the reagents and their concentrations for each reference. In this stage, the cell biomass growth (g/L), acid phosphatase activity (U/mL), and pH were evaluated for each of the three fungi.

### Effect of phosphate sources

As shown in Table 2, three phosphate sources were evaluated in Tr1, Tr2, and Tr3 culture media for the three microorganisms studied. The objective of this assay was to evaluate the production of acid phosphatase in relation to the availability of a soluble phosphate source in the culture medium. CaHPO₄ has low solubility and was therefore used at a higher concentration. KH₂PO₄ shows intermediate solubility, whereas NaH₂PO₄ is highly soluble in aqueous media. Submerged fermentations were carried out under the conditions described in Sect.  2.6. Acid phosphatase activity was determined according to the method described in Sect.  2.5. The enzyme activity values used for statistical analysis correspond to the highest activity observed during fermentation for each experimental condition. A two-way analysis of variance (ANOVA) with replication was performed at a significance level of α = 0.05 to evaluate differences among treatments.

**Table 2:** Concentration of the reagents used in the experiment

Treatments	Sugar (g/L)	Yeast extract (g/L)	Source of phosphate (g/L)
Tr1(1)	20.0	5.0	5.0
Tr2(2)	20.0	5.0	2.0
Tr3(3)	20.0	5.0	1.0

Tr_1_(1): Culture medium with CaHPO_4_ as the phosphate source. Tr_2_(2): Culture medium with KH_2_PO_4_ as the phosphate source. Tr_3_(3): Culture medium with NaH_2_PO_4_ as the phosphate source

### Optimization of the culture medium for acid phosphatase production by *Trichoderma spp.* strains

After defining the modified JP culture medium, a Central Composite Design (CCD) was performed to evaluate the effects of reagent concentrations on fungal growth. The design included 2³ factorial points, 3 replicates at the central point, and 6 axial points, totaling 17 experimental runs, which were conducted in duplicate (34 runs in total). The CCD was applied separately for each *Trichoderma* strain studied. The variables investigated were: (X₁) sucrose, (X₂) industrial yeast extract (Biorigin), and (X₃) monobasic potassium phosphate. The responses measured were phosphatase activity, cell biomass (dry mass, g/L), and substrate consumption. The rotatability coefficient (α) was 1.682. The actual and coded values of each variable are presented in Table 3, based on preliminary tests. Statistical analyses were performed using Statistica 7.1 (StatSoft).

**Table 3:** Real and coded values of the analyzed variable

Variable	–α	–1	0	+ 1	+α
Sucrose – X1 (g/L)	6.6	10.0	15.00	20.0	23.40
Yeast extract – X2 (g/L)	0.00	2.00	5.00	8.00	10.00
Monobasic potassium phosphate – X3 (g/L)	0.16	0.50	1.00	1.50	1.84

### Lyophilization of enzymes

Crude enzyme extracts were dialyzed to remove salts using regenerated cellulose membranes (12–14 kDa molecular weight cut-off) against distilled water at 4 °C for 24 h, with periodic water replacement every 6 h. After dialysis, the samples were frozen at − 80 °C and lyophilized in a bench-top freeze dryer (Liotop L101, Brazil) according to the manufacturer’s instructions (condenser temperature − 50 °C and pressure below 0.1 mbar). The lyophilized enzymes were stored at − 20 °C until further use.

### Enzyme profile analysis by SDS-PAGE

For SDS-PAGE analysis, protein samples were optionally concentrated by trichloroacetic acid (TCA) precipitation when required. Briefly, 225 µL of 100% (w/v) TCA was added to 900 µL of the enzyme extract, and the mixture was incubated at 4 °C overnight. Samples were centrifuged at 20,000 × g for 15 min, and the resulting pellets were washed three times with ice-cold 100% acetone, with centrifugation at 20,000 × g for 15 min after each wash. After drying, pellets were resuspended in 20 µL of 1× sample buffer.

Protein samples were denatured at 100 °C for 5 min and separated by SDS-PAGE using 12% (w/v) polyacrylamide gels prepared according to the method described by Laemmli (1970). Electrophoresis was carried out in Tris–glycine–SDS running buffer (25 mM Tris, 192 mM glycine, and 0.1% SDS) at a constant voltage of 100 V. Protein bands were visualized by Coomassie Brilliant Blue staining. When necessary, silver staining was performed to detect low-abundance proteins. Molecular weight estimation was performed using a commercial protein marker ranging from 10 to 250 kDa.

### Optimization of reagents in the selected medium by Central Composite Design in submerged fermentation

Treatments were performed in a shaker incubator as described in Sect.  2.8. The Central Composite Design (CCD) was developed to maximize acid phosphatase production by the 3 *Trichoderma* sp. strains. This design was proposed at 3 levels, with 3 repetitions at the central point, and 3 variables were studied: X1 – sucrose; X2 – industrial yeast extract from Biorigin brand; and X3 – monobasic potassium phosphate. All treatments were performed in duplicates, totaling 34 treatments for each strain.

The analyzed response was acid phosphatase activity (ACPase) expressed in (U/mL) for each microorganism at 72 h, the final batch time. All statistical data were calculated using StatSoft Software Statistica 7.1 and are shown in Table 4.

**Table 4:** Concentrations of the variables proposed by the Central Composite Design and response results at 72 h for each microorganism

Assays	X1 (g/L)	X2 (g/L)	X3 (g/L)	ACPase (1) T.a (U/mL)	ACPase (2) T.h (U/mL)	ACPase (3) T.*r* (U/mL)
1	(-1)10.00	(-1)2.00	(-1)0.50	0.133	0.584	0.830
2	(-1)10.00	(-1)2.00	(+ 1)1.50	0.082	0.452	1.146
3	(-1)10.00	(+ 1)8.00	(-1)0.50	0.589	0.620	1.116
4	(-1)10.00	(+ 1)8.00	(+ 1)1.50	0.464	0.480	2.024
5	(+ 1)20.00	(-1)2.00	(-1)0.50	0.138	0.676	1.279
6	(+ 1)20.00	(-1)2.00	(+ 1)1.50	0.090	0.494	1.361
7	(+ 1)20.00	(+ 1)8.00	(-1)0.50	0.530	0.715	1.942
8	(+ 1)20.00	(+ 1)8.00	(+ 1)1.50	0.591	0.560	2.790
9	(-α)6.60	(0)5.00	(0)1.00	0.261	0.520	1.035
10	(+α)23.41	(0)5.00	(0)1.00	0.422	0.964	2.538
11	(0)15.00	(-α)0.00	(0)1.00	0.058	0.052	0.085
12	(0)15.00	(+α)10.5	(0)1.00	0.401	0.249	1.822
13	(0)15.00	(0)5.00	(-α)0.16	0.650	0.738	1.476
14	(0)15.00	(0)5.00	(+α)1.84	0.255	0.308	2.556
15	(0)15.00	(0)5.00	(0)1.00	0.452	1.006	2.010
16	(0)15.00	(0)5.00	(0)1.00	0.463	1.033	2.144
17	(0)15.00	(0)5.00	(0)1.00	0.436	0.997	2.138

ACPase T.a (U/mL): Acid phosphatase activity for *T. asperellum*

ACPase T.h (U/mL): Acid phosphatase activity for *T. harzianum*

(3) ACPase T.r (U/mL): Acid phosphatase activity for *T. reesei*

## Results and discussion

###  Morphology of fungi by SEM

**Fig. 1:**
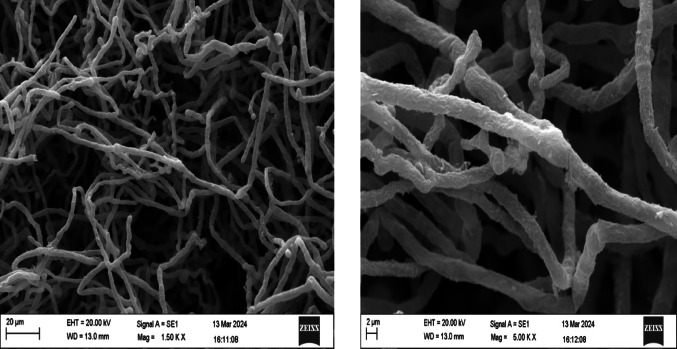
Scanning electron microscopy (SEM) images of *T. asperellum* grown under submerged fermentation conditions, showing septate and cylindrical hyphae with a dense and compact mycelial structure. Scale bars are indicated in each image

**Fig. 2:**
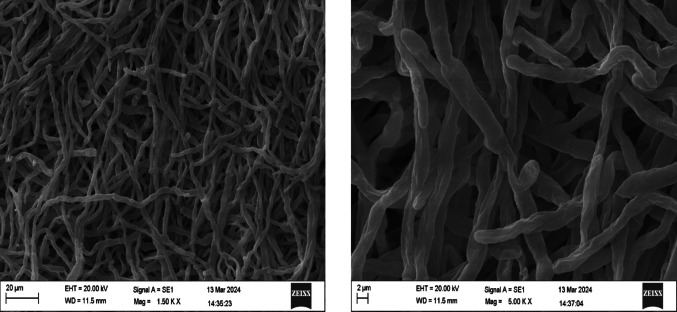
Scanning electron microscopy (SEM) images of *T. reesei* grown under submerged fermentation conditions, showing thinner and less-branched hyphae compared to *T. asperellum*. Scale bars are indicated in each image

Scanning electron microscopy (SEM) was performed to qualitatively evaluate the morphological characteristics of *Trichoderma spp.* under submerged fermentation conditions. The analysis was conducted for *T. asperellum* and *T. reesei* as representative strains, aiming to provide complementary information on hyphal organization and growth patterns in liquid culture. SEM observations confirmed the filamentous morphology of both species, with septate and cylindrical hyphae, although differences in mycelial organization were observed between the strains (Figs. 1 and 2).

At higher magnifications (20,000×), *T. asperellum* presented a denser and more compact hyphal network, whereas *T. reesei* showed thinner and less-branched hyphae. These observations provide qualitative information on fungal growth behavior under submerged fermentation conditions. No direct causal relationship between fungal morphology and acid phosphatase activity can be inferred from SEM analysis alone, and the results are presented as complementary morphological characterization without functional or mechanistic implications.

### Acid phosphatase production potential by submerged fermentation in different culture media

The potential for ACPase production in 6 culture media was analyzed at 0, 24, 48, and 72 h during submerged fermentation. Additionally, cellular biomass production (g/L) was quantified, and pH was monitored at all sampling points. Experiments were performed in triplicate, and the results for the last sampling point (72 h of process) are represented in Table 5 along with the Tukey test.

**Table 5:** Acid phosphatase production potential, cellular biomass, and final pH in synthetic culture media

Culture Medium*	Cellular Biomass (g/L)	ACPase Activity (U/mL)	pH	References
*T. asperellum*				
MC-1	8.30 ± 0.21	0.151 ± 0.006 (b, C)	4.36 ± 0.16	Laboratory Farroupilha
MC-2	4.45 ± 0.44	0.314 ± 0.019 (a, C)	4.25 ± 0.06	Laboratory Farroupilha/UFU
MC-3	3.84 ± 0.13	0.166 ± 0.003 (b, C)	5.05 ± 0.14	Adapted Araújo (2014)
MC-4	2.47 ± 0.22	0.059 ± 0.012 (c, D)	2.53 ± 0.02	Sampaio et al., (2003)
MC-5	3.10 ± 0.06	0.079 ± 0.006 (c, D)	6.93 ± 0.06	Leitão et al. (2010)
MC-6	6.32 ± 0.14	0.085 ± 0.006 (c, D)	2.02 ± 0.03	Souza et al. (2016)
*T. harzianum*				
MC-1	7.33 ± 0.54	0.199 ± 0.006 (b, B)	5.01 ± 0.06	Laboratory Farroupilha
MC-2	4.46 ± 0.20	0.647 ± 0.050 (a, A)	5.50 ± 0.08	Laboratory Farroupilha/UFU
MC-3	3.71 ± 0.34	0.143 ± 0.015 (bc, C)	5.16 ± 0.09	Adapted Araújo (2014)
MC-4	3.68 ± 0.12	0.062 ± 0.006 (d, D)	2.05 ± 0.02	Sampaio et al. (2003)
MC-5	3.00 ± 0.06	0.067 ± 0.006 (d, D)	6.00 ± 0.21	Leitão et al. (2010)
MC-6	4.76 ± 0.56	0.095 ± 0.022 (cd, D)	2.82 ± 0.02	Souza et al. (2016)
*T. reesei*				
MC-1	7.70 ± 0.10	0.137 ± 0.012 (c, C)	4.31 ± 0.16	Laboratory Farroupilha
MC-2	6.09 ± 0.09	0.550 ± 0.016 (a, B)	5.37 ± 0.09	Laboratory Farroupilha/UFU
MC-3	4.34 ± 0.09	0.226 ± 0.015 (b, B)	4.99 ± 0.18	Adapted Araújo (2014)
MC-4	3.01 ± 0.18	0.049 ± 0.007 (e, D)	2.34 ± 0.04	Sampaio et al. (2003)
MC-5	2.02 ± 0.09	0.079 ± 0.012 (de, D)	6.99 ± 0.08	Leitão et al. (2010)
MC-6	6.49 ± 0.64	0.091 ± 0.016 (d, D)	2.33 ± 0.09	Souza et al. (2016)

(*) Culture medium: The tested culture media are defined in Sect.  3.3. Lowercase letters: Tukey test analyzing different culture media with the same microorganism. Uppercase letters: Tukey test analyzing different microorganisms for each culture medium

These results indicate that the three best culture media were MC-1, MC-2, and MC-3, with emphasis on MC-1, which showed the highest cellular biomass growth for all tested microorganisms, ranging from 7.33 to 8.30 (g/L). This medium also presented the second-best result for ACPase activity, which ranged from approximately 0.137 to 0.199 (U/mL). The results for cellular biomass growth were expected because this medium is used in the Farroupilha Biocontrol Laboratory to develop *T. asperellum* hyphae for the Quality product.

Culture medium MC-2 was an adaptation of MC-1 to which potassium hydrogen phosphate (KH₂PO₄) was added. This salt is widely used as a food additive and fertilizer and is an excellent source of potassium and phosphorus. It is also a buffering agent. The best results for enzymatic activity in this assay were for this culture medium, reaching values of approximately 0.647 (U/mL) for *T. harzianum*, more than triple the value found in MC-1 for the same microorganism. The final cellular biomass in this culture medium that stood out most was 6.09 ± 0.09 (g/L) for *T. reesei*.

Another noteworthy culture medium is MC-4. It was used to select phosphate-solubilizing microorganisms in Cabral’s (2016) work. It was responsible for selecting *T. harzianum* (microorganism identified by Fundação André Tosello de Pesquisa e Tecnologia, Campinas/SP) in that study. Notably, after 72 h of fermentation, the pH values for the *Trichoderma strains* ranged from 2.05 to 2.53. Although such acidic conditions negatively affected enzyme production, resulting in negligible acid phosphatase activity, this behavior has also been reported by Cabral (2016), who attributed the pH reduction to the formation of acetic and lactic acids. In the present study, these conditions favored phosphate rock solubilization.

Culture media MC-5 and MC-6 did not yield satisfactory results for enzymatic activity, although MC-6 showed good cellular biomass development for all microorganisms. Leitão et al. (2010), studying ACPase secreted by *T. harzianum* fermenting MC-5 medium, identified a specific activity of 12.4 (U/mg) in a pH range varying from 4.5 to 6.0. Souza et al. (2016), using MC-6 culture medium and inoculating 107 (spores/mL) of *T. harzianum*, with pH adjusted to 4.0, obtained 14.3 (U/mg) of specific activity in the crude extract after 48 h.

Similar effects of culture medium composition on enzyme production have been reported in recent studies, emphasizing the importance of nutrient balance for optimizing fungal metabolism and enzymatic expression under submerged fermentation conditions (Greco-Duarte et al. 2023).

Therefore, the synthetic culture medium selected for the optimization stage was MC-2. This medium presents the highest average ACPase activity for all microorganisms and differs significantly from the other culture media according to the Tukey test. Subsequent steps in evaluating phosphate sources and optimizing reagents using the CCD for each microorganism were performed.

### Evaluation of phosphate sources for acid phosphatase production in submerged fermentation

Table 6 shows the results of the statistical analysis regarding the enzymatic activity response at 48 h and 72 h for *T. asperellum*, *T. harzianum*, and *T. reesei*. The analysis of the culture media fermented by *T. asperellum* showed a p-value of 0.002, much lower than the α value of 0.05 adopted in the analysis, indicating that treatments Tr1, Tr2, and Tr3 have a statistical difference among them. Also, the F-value being greater than the calculated F leads to the same conclusion. Regarding the fermentation times for this microorganism, the p-value is also lower than the adopted α, which leads to the conclusion that the values are different among them, and this indicates that it has not yet reached the stationary state, making it interesting to ferment for longer. This same reasoning can be obtained by analyzing the F-value being greater than F.

**Table 6:** ANOVA Table for enzymatic activity of the three microorganisms at 48 and 72 h

Source of variation	SQ	DF	MQ	F	*p*-value	F-critical
Samples Tr1, Tr2 and Tr3 – Enzymatic Activity (*T. asperellum*)	0.014	2	0.007	21.050	0.002	5.143
Fermentation time 48 h and 72 h (*T. asperellum*)	0.009	1	0.009	25.986	0.002	5.987
Samples Tr1, Tr2 and Tr3 – Enzymatic Activity (*T. harzianum*)	0.171	2	0.085	44.591	2.50 × 10⁻⁴	5.143
Fermentation time 48 h and 72 h (*T. harzianum*)	5.50 × 10⁻⁴	1	5.50 × 10⁻⁴	0.271	0.621	5.987
Samples Tr1, Tr2 and Tr3 – Enzymatic Activity (*T. reesei*)	0.095	2	0.047	17.004	0.003	5.143
Fermentation time 48 h and 72 h (*T. reesei*)	0.002	1	0.002	0.682	0.440	5.987

The statistical analysis for the enzymatic activity of *T. harzianum* and *T. reesei* led to the same conclusions. For both microorganisms, the p-values associated with treatments Tr1, Tr2, and Tr3 were much lower than the adopted significance level (α = 0.05), indicating statistically significant differences among treatments. In contrast, the p-values related to fermentation time were higher than α, indicating no significant variation in enzymatic activity between 48 and 72 h. These results are consistent with the F-test, as the calculated F-values were lower than the corresponding critical F-values.

**Fig. 3:**
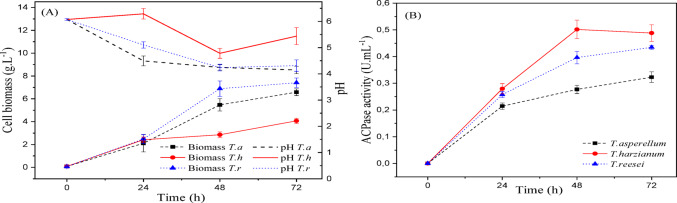
(a) Cellular biomass (g/L) and pH variation; (b) acid phosphatase activity (U/mL) during 72 h of submerged fermentation of *Trichoderma spp.* cultivated in Tr1 medium (CaHPO₄ as phosphate source) at 25 °C and 150 rpm. Data represent mean ± standard deviation (*n* = 3)

**Fig. 4:**
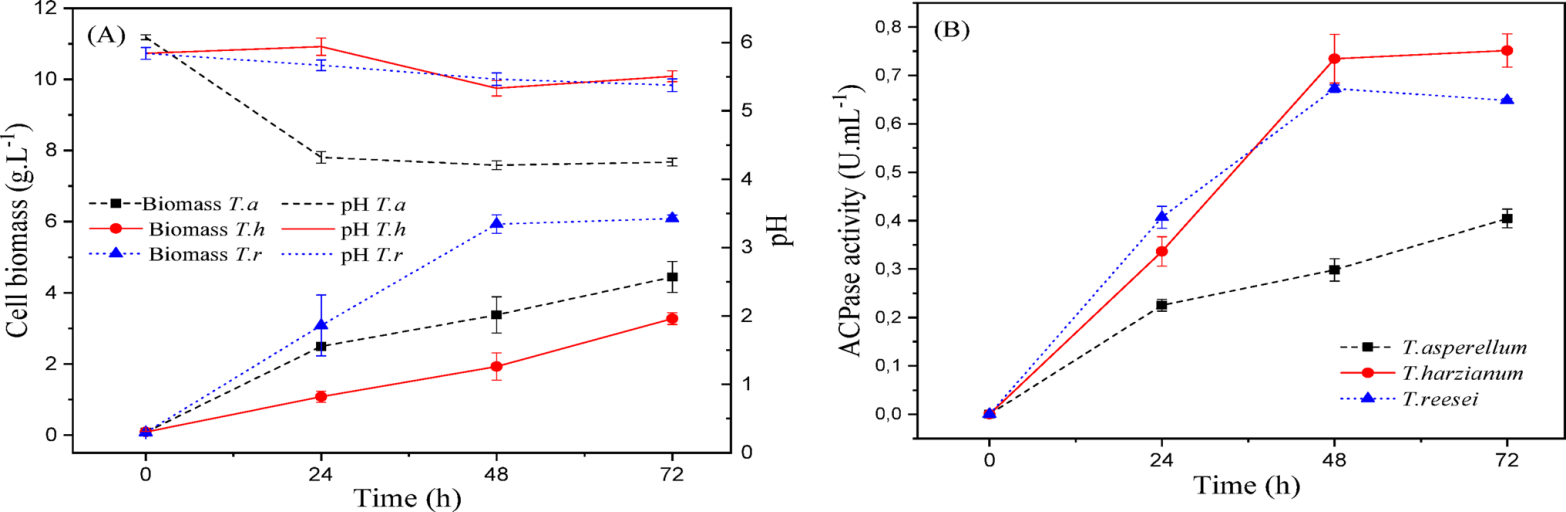
(a) Cellular biomass (g/L) and pH variation; (b) acid phosphatase activity (U/mL) during 72 h of submerged fermentation of *Trichoderma spp.* cultivated in Tr2 medium (KH₂PO₄ as phosphate source) at 25 °C and 150 rpm. Data represent mean ± standard deviation (*n* = 3)

**Fig. 5:**
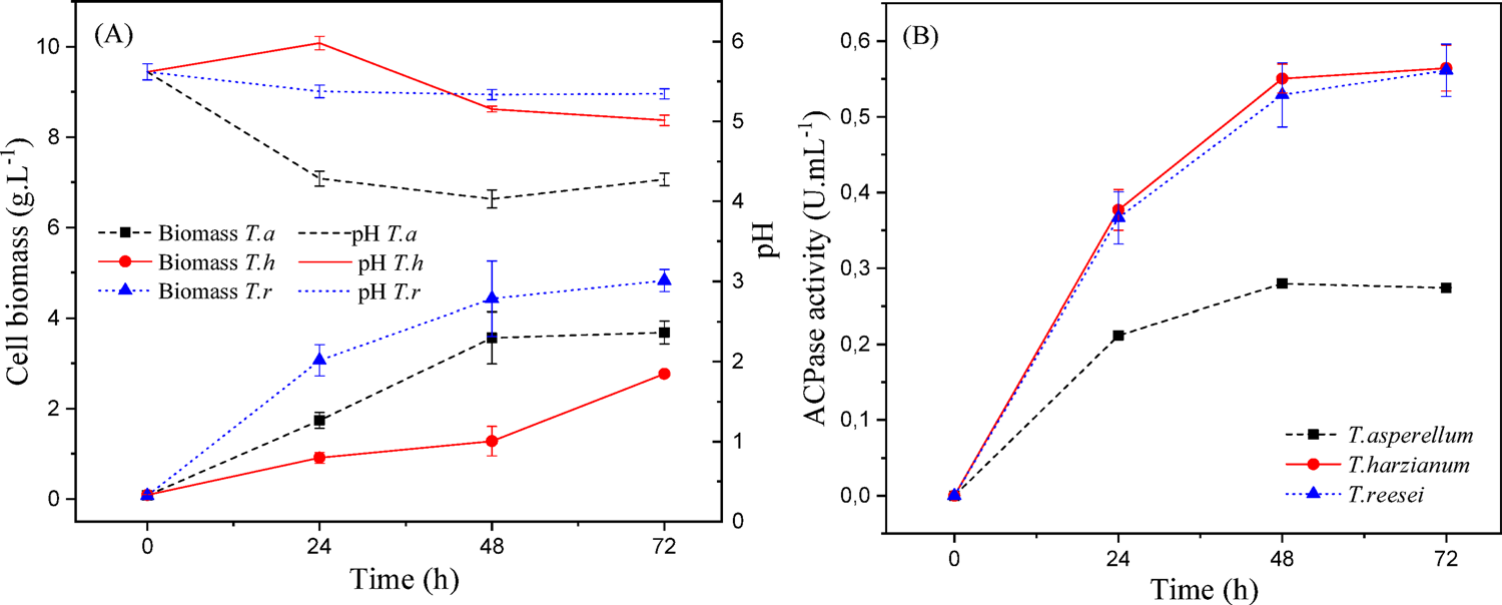
(a) Cellular biomass (g/L) and pH variation; (b) acid phosphatase activity (U/mL) during 72 h of submerged fermentation of *Trichoderma spp.* cultivated in Tr3 medium (NaH₂PO₄ as phosphate source) at 25 °C and 150 rpm. Data represent mean ± standard deviation (*n* = 3)

Figures 3 and 4, and 5 show the kinetics of cellular growth (dry biomass), acid phosphatase activity, and pH variation over 72 h of liquid fermentation for culture media Tr1, Tr2, and Tr3, respectively.

Through the analysis of the Figures, it is noted that *T. reesei* showed better growth in all studied phosphate sources. After 72 h of batch, the highest dry biomass for this microorganism was 7.41 (g/L), in Tr_1_ culture medium, which has calcium phosphate as a phosphorus source.

It can be observed in Fig. 5 (b) that *T. harzianum* proved to be more promising for enzyme production under analysis, compared to the others. In this Tr_2_ culture medium, supplemented with KH_2_PO_4_, this microorganism reached its maximum value of 0.672 (U/mL) of activity in the first 48 h, then showed little variation until the end of fermentation. The second-best result was for *T. reesei*, showing 16.8% lower enzymatic activity than *T. harzianum* and an average final dry biomass of 6.09 (g/L) for this culture medium.

The regulatory effect of phosphate availability on phosphatase production has also been described in recent studies, where high phosphate concentrations may repress enzyme synthesis due to feedback regulation mechanisms (Abdelgalil et al. 2022). Similarly, Leitão et al. (2010) reported acid phosphatase production by *T. harzianum* under submerged fermentation, achieving a specific activity of 12.4 (U/mg) and an overall yield of 56.3%.

Studies on acid phosphatase production by *T. asperellum* Q1 under saline stress conditions positively impacted *Arabidopsis* growth (Zhao et al. 2017). These researchers also studied phosphorus solubilization in 3 phosphate sources: tricalcium phosphate (TCP), dibasic calcium phosphate (DCP), and phytic acid in the presence and absence of salt. The best enzymatic production was 9.61.10^− 3^ (U/mL) for culture medium containing phytic acid as a P source to activate acid phosphatase production. Subsequently, they purified this enzyme and applied it to promote *Arabidopsis* growth.

It is important to note that there was no pH correction for any treatment, and all values were less than 6.0 (pH in the acidic range) throughout the fermentation process.

Therefore, the phosphate source influenced the production of the acid phosphatase enzyme, and KH_2_PO_4_ was the phosphate source chosen to be optimized in future steps.

### Evaluation of acid phosphatase production by *T. asperellum* with supplementation of the medium proposed by CCD

The experimental data obtained from the Central Composite Design were fitted to a second-order polynomial model. The linear effects of the independent variables on acid phosphatase activity are described by Eq. (1).

1 $$\begin{aligned} {\text{ACPase }}\left[ {{\mathrm{T}}.{\mathrm{a}}} \right]{\text{ }}\left( {{\mathrm{U}}/{\mathrm{mL}}} \right)\, = & 0.{\mathrm{45}}\, + \,0.0{\text{2 X1}}{-}0.0{\mathrm{4}}0{\text{ X12}}\, \\ & + \,0.{\text{169 X2}}{-}0.0{\text{79 X22}} \\ & {-}0.{\text{61 X3}}\, + \,0.0{\text{24 X1 X3}} \\ \end{aligned}$$

The coefficient of determination (R^2^) was 0.86, indicating that approximately 86% of the experimental data are described by the model proposed in Eq. 1. The negative value of the linear coefficient of X3 shows that higher concentrations of monobasic potassium phosphate decrease the activity response for this microorganism. This can be observed in Table 4 by comparing treatments 1 and 2 or also 3 and 4, for example.

The variable X2 has the greatest effect in this equation due to having the largest linear coefficient, meaning higher concentrations of it increase enzymatic activity. This can also be exemplified by comparing the results of treatments 2 and 4 in Table 4.

**Fig. 6:**
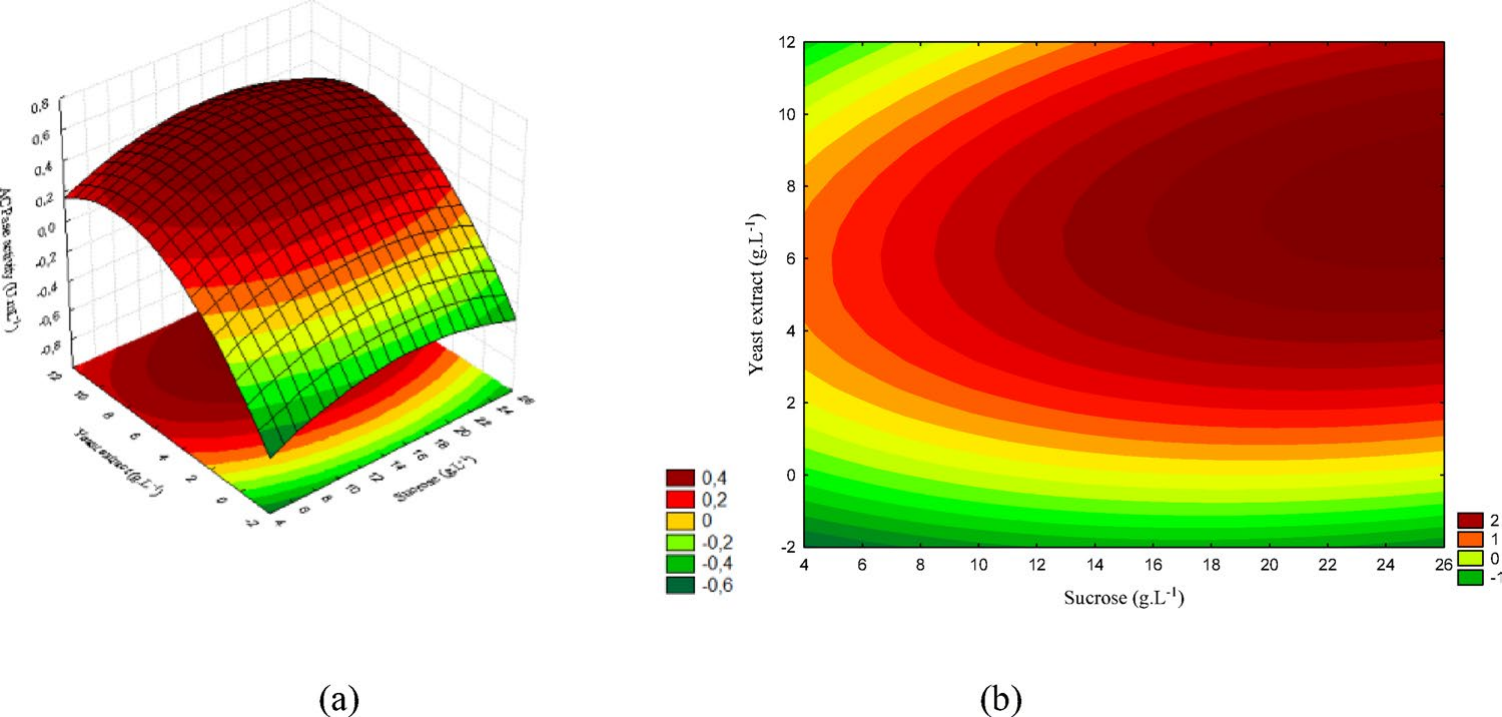
(a) Response surface and (b) contour plot showing the effect of sucrose and yeast extract concentrations (g/L) on acid phosphatase activity (U/mL) for *T. asperellum*. The concentration of KH₂PO₄ was kept constant at the central level

**Fig. 7:**
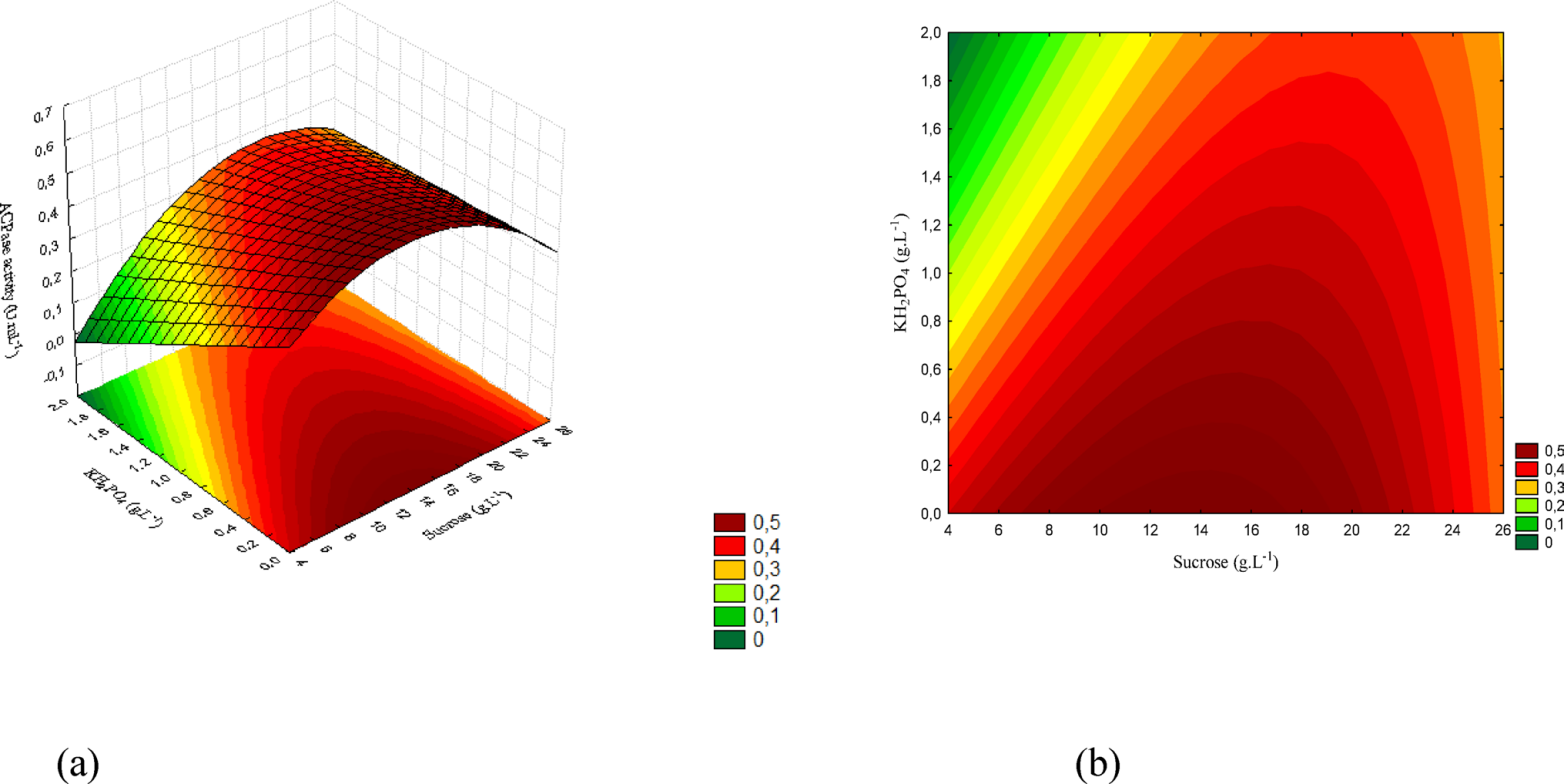
(a) Response surface and (b) contour plot showing the effect of sucrose and KH₂PO₄ concentrations (g/L) on acid phosphatase activity (U/mL) for *T. asperellum*. The concentration of yeast extract was kept constant at the central level

**Fig. 8:**
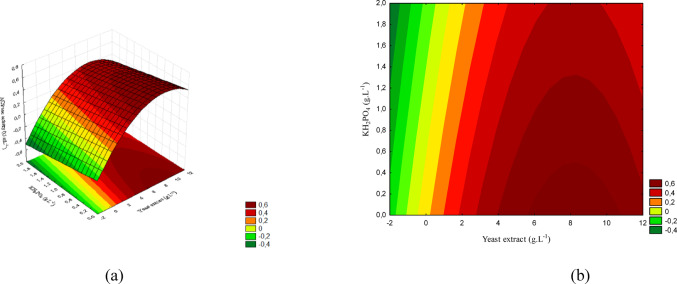
(a) Response surface and (b) contour plot showing the effect of yeast extract and KH₂PO₄ concentrations (g/L) on acid phosphatase activity (U/mL) for *T. asperellum*. The sucrose concentration was kept constant at the central level

The results observed for these microorganisms are presented in Figs. 6(a) and (b), 7(a) and (b), and 8(a) and (b).

The concentration ranges corresponding to the response surface are presented in Fig. 6 (a), while Fig. 6 (b) shows the contour plot. The graphs represent the interaction between sucrose and yeast extract. The highest acid phosphatase activity was obtained at intermediate sucrose concentrations (11–17.5 g/L) combined with high levels of yeast extract (6–10.5 g/L), reaching values close to 0.65 (U/mL).

Figure 7 (a) presents the response surface, and Fig. 7 (b) the contour plot for the interaction between sucrose and KH₂PO₄. The maximum activity was obtained at low KH₂PO₄ concentrations (< 0.2 g/L), even under favorable sucrose levels. Under these conditions, the activity reached values close to the maximum predicted for this microorganism.

The concentration ranges corresponding to the response surface are shown in Fig. 8 (a), whereas Fig. 8 (b) presents the contour plot. The interaction between yeast extract and KH₂PO₄ demonstrates that the highest activity was achieved at high yeast extract levels and low KH₂PO₄ concentrations, confirming the inhibitory effect of excess phosphorus on enzyme production.

### Evaluation of acid phosphatase production by *T. harzianum* with supplementation of the medium proposed by CCD

The interaction effects between the independent variables were evaluated according to Eq. (2), which represents the combined influence of the studied factors on enzyme activity.

2 $$\begin{aligned} \:ACPase\left[ {T.a} \right]\left( {\frac{U}{{mL}}} \right)\: = \: & \:1.003\: + \:0.077\:X1\: - \:0.066\:X12\: \\ & + \:0.037\:X2\: - \:0.275\:X22\: - \\ & \:0.097\:X3\: + \:0.006\:X1\:X3\: \\ \end{aligned}$$

The coefficient of determination (R^2^) was 0.92, indicating that approximately 92% of the experimental data are described by the model proposed in Eq. 2. The positive values of the linear coefficients X1 and X2 show that higher concentrations of sucrose and yeast extract favor enzyme production, as their activity increases. This behavior can be seen through treatments 2 and 8 in Table 4.

The negative value of the linear coefficient of X3 shows that higher concentrations of monobasic potassium phosphate decrease the activity response for this microorganism. This can be observed in Table 4 by comparing treatments 5 and 6 or also 7 and 8, for example.

The variable X3 also has the greatest effect in this equation due to having the largest linear coefficient. However, this effect is unfavorable, meaning higher concentrations of it result in lower enzymatic activity. This can also be exemplified by comparing the results of treatments 1 and 2 in Table 4.

**Fig. 9:**
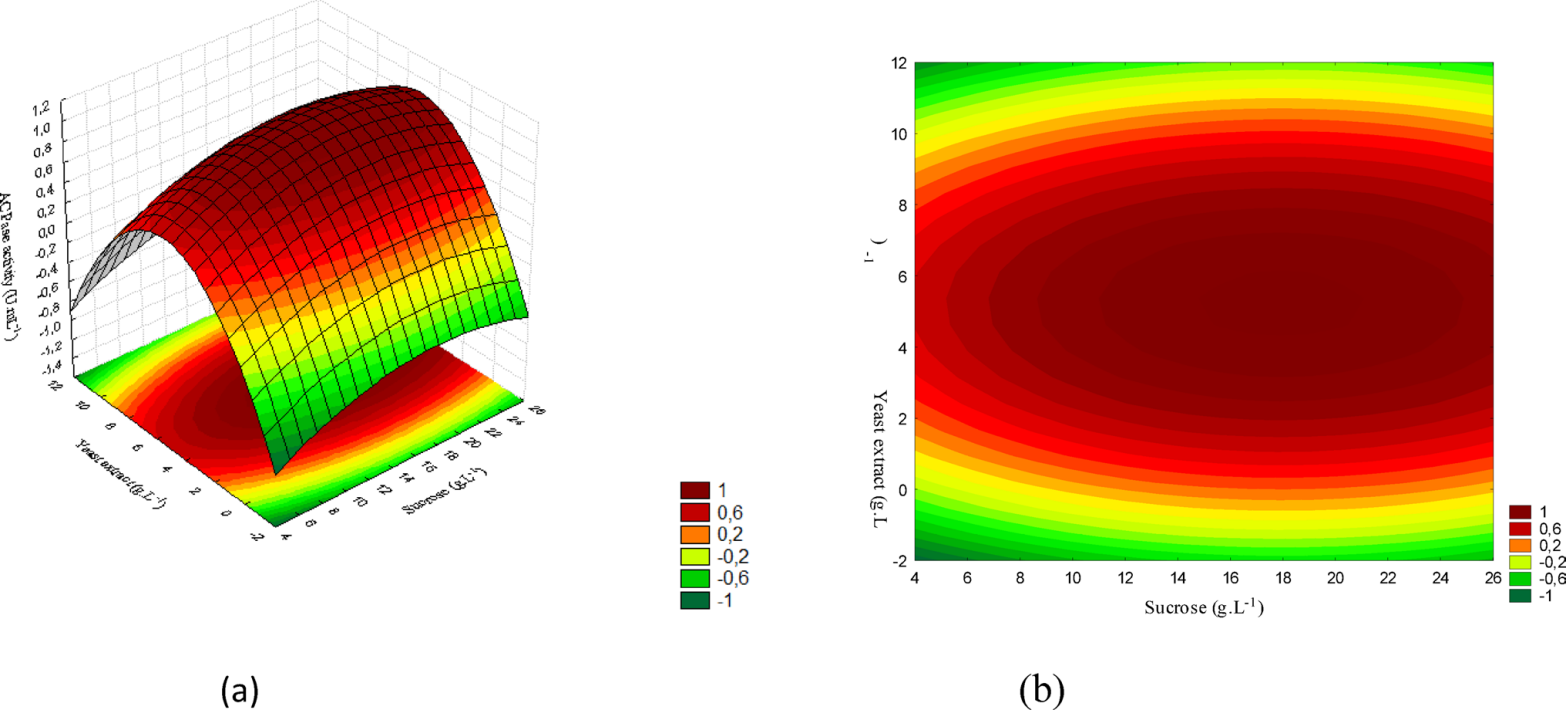
(a) Response surface and (b) contour plot showing the effect of sucrose and yeast extract concentrations (g/L) on acid phosphatase activity (U/mL) for *T. harzianum*. The concentration of KH₂PO₄ was kept constant at the central level

**Fig. 10:**
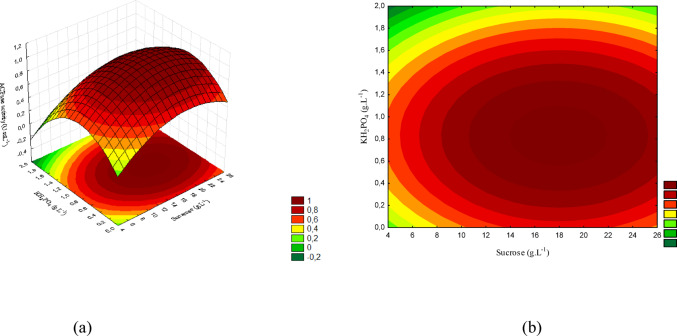
(a) Response surface and (b) contour plot showing the effect of sucrose and KH₂PO₄ concentrations (g/L) on acid phosphatase activity (U/mL) for *T. harzianum*. The concentration of yeast extract was kept constant at the central level

**Fig. 11:**
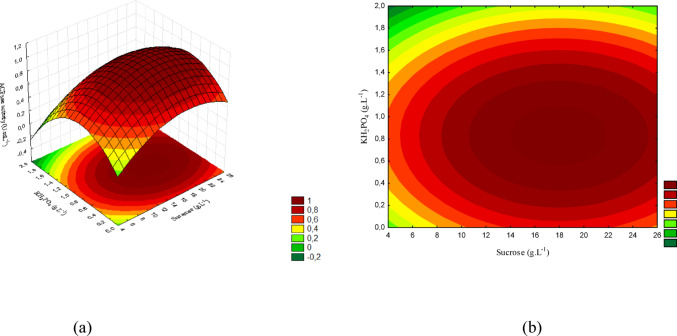
(a) Response surface and (b) contour plot showing the effect of yeast extract and KH₂PO₄ concentrations (g/L) on acid phosphatase activity (U/mL) for *T. harzianum.* The sucrose concentration was kept constant at the central level

The results observed for these microorganisms are presented in Figs. 9 (a) and (b), 10 (a) and (b), and 11 (a) and (b).

Figure 9 (a) shows the response surface and Fig. 9 (b) the contour plot for the interaction between sucrose and yeast extract. The highest activities were obtained at intermediate to high concentrations of both factors (sucrose 14.5–22 g/L and yeast extract 3.5–7 g/L), reaching maximum values of approximately 1.0 (U/mL).

The concentration ranges corresponding to the response surface are displayed in Fig. 10 (a), whereas Fig. 10 (b) shows the contour plot. The interaction between sucrose and KH₂PO₄ indicates that increasing KH₂PO₄ reduces activity, even at favorable sucrose levels. The maximum response occurred at low KH₂PO₄ concentrations associated with intermediate sucrose levels.

Figure 11 presents the response surface and the contour plot for the interaction between yeast extract and KH₂PO₄. The maximum activity was obtained at high yeast extract levels combined with low KH₂PO₄ concentrations, confirming the inhibitory effect of excess phosphorus on this strain.

### Evaluation of acid phosphatase production by *T. reesei* with supplementation of the medium proposed by CCD

Finally, the complete quadratic model, including linear, interaction, and squared terms, is expressed by Eq. (3), which was used to predict acid phosphatase activity within the experimental domain.

3 $$\begin{aligned} {\mathrm{ACPase}}\left[ {{\mathrm{T}}.{\mathrm{r}}} \right]{\text{ }}\left( {{\mathrm{U}}/{\mathrm{mL}}} \right)\, = & \,{\mathrm{2}}.0{\mathrm{62}}\, + \,0.{\text{348 X1}}{-}0.0{\text{97 X1}}^{{\mathrm{2}}} \, \\ & + \,0.{\text{456 X2}}{-}0.{\text{399 X2}}^{{\mathrm{2}}} \, + \,0.{\text{291 X3}}\, \\ & + \,0.{\text{116 X1 X2}}\, + \,0.{\mathrm{17}}0{\text{ X2 X3}} \\ \end{aligned}$$

The coefficient of determination (R^2^) was 0.97, indicating that approximately 97% of the experimental data are described by the model proposed in Eq. 3. The positive values of the linear coefficients X1, X2, and X3 show that higher concentrations of sucrose, yeast extract, and monobasic potassium phosphate favor enzyme production, as its activity increases. This behavior can be exemplified through treatments 1 and 8 or 1 and 15 in Table 4. In the first example, the increase in the concentration of significant variables increased enzymatic activity by 336%, i.e., more than 3 times.

**Fig. 12:**
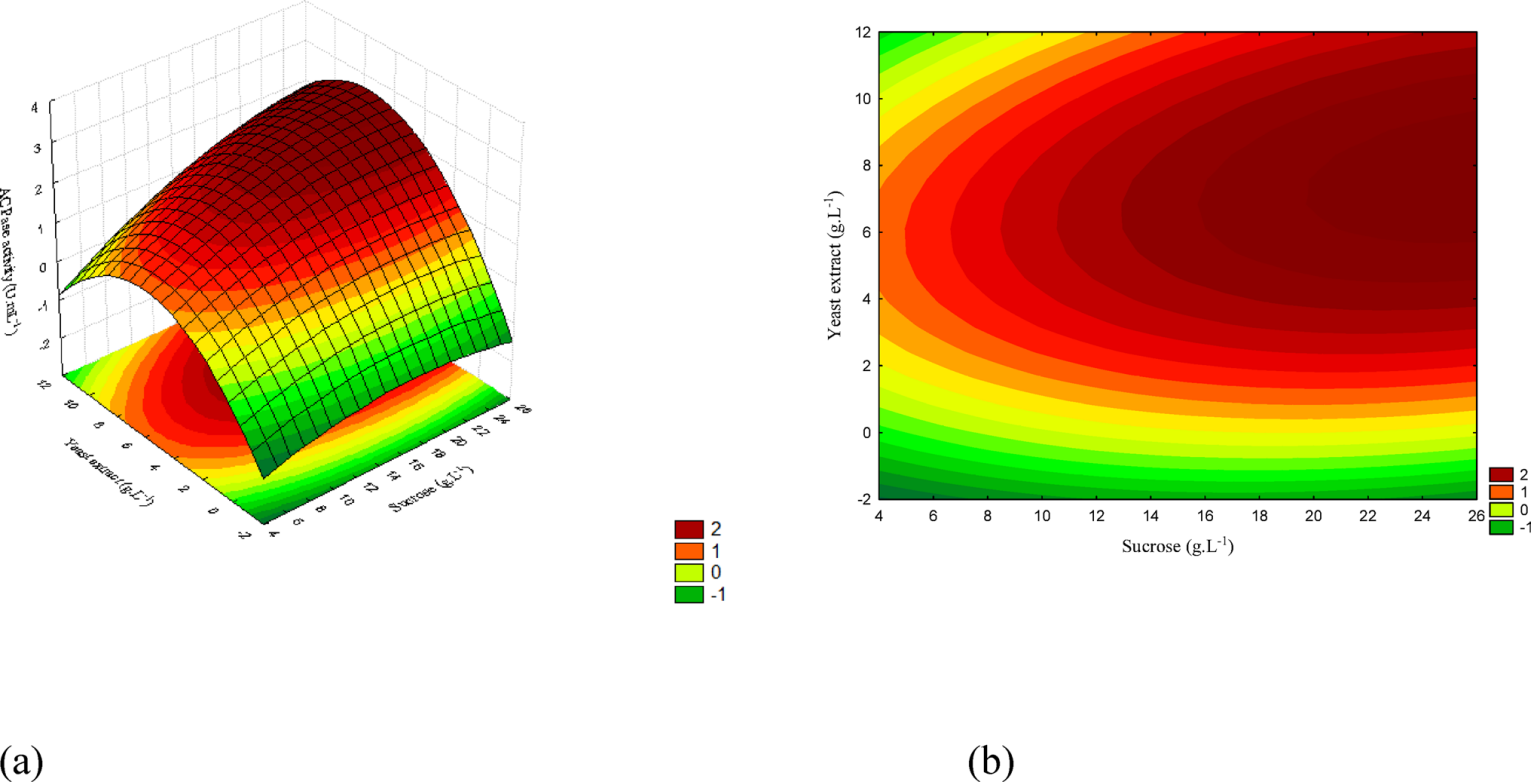
(a) Response surface and (b) contour plot showing the effect of sucrose and yeast extract concentrations (g/L) on acid phosphatase activity (U/mL) for *T. reesei*. The concentration of KH₂PO₄ was kept constant at the central level

**Fig. 13:**
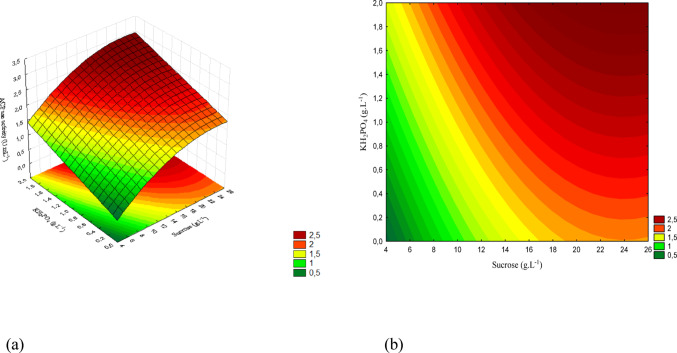
(a) Response surface and (b) contour plot showing the effect of sucrose and KH₂PO₄ concentrations (g/L) on acid phosphatase activity (U/mL) for *T. reesei*. The concentration of yeast extract was kept constant at the central level

**Fig. 14:**
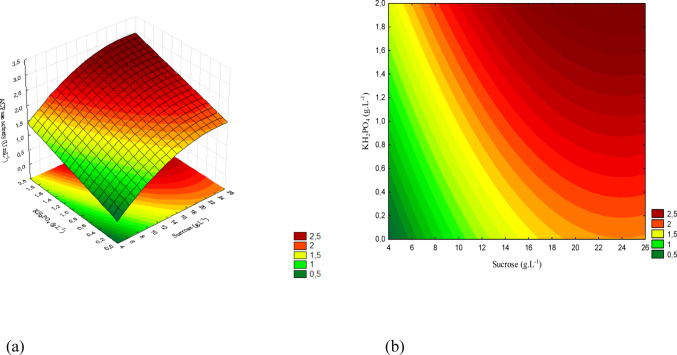
(a) Response surface and (b) contour plot showing the effect of yeast extract and KH₂PO₄ concentrations (g/L) on acid phosphatase activity (U/mL) for *T. reesei*. The sucrose concentration was kept constant at the central level

The concentration ranges are presented in Figs. 12(a), 13(a), and 14(a), while Figs. 12(b), 13(b), and 14(b) show the corresponding contour plots. Each plot illustrates the pairwise interactions among the variables of interest.

The concentration ranges corresponding to the response surface are shown in Fig. 12 (a), while Fig. 12 (b) presents the contour plot. The interaction between sucrose and yeast extract demonstrates that activity increased progressively with higher concentrations of both factors, reaching values close to 2.8 (U/mL).

Figure 13 presents the response surface and the contour plot for the interaction between sucrose and KH₂PO₄. The highest activity was observed at elevated concentrations of both factors, indicating a positive effect of phosphorus when combined with higher sucrose levels.

The concentration ranges corresponding to the response surface are displayed in Fig. 14 (a), while Fig. 14 (b) shows the contour plot. The interaction between yeast extract and KH₂PO₄ demonstrates that the highest activity values were obtained at high levels of both factors, confirming the synergistic effect of these nutrients for *T. reesei*.

Therefore, the Central Composite Design improved the enzymatic production of all microorganisms when compared to preliminary tests. The liquid fermentation of *T. harzianum* showed the worst results among the three fungal strains. In initial tests, it had an average enzymatic activity of 0.750 (U/mL), and after the CCD, the acid phosphatase activity reached a maximum value of 1.006 (U/mL), i.e., 34% higher than preliminary tests.

The best comparative results were obtained for *T. reesei*. Previously, this strain showed an average acid phosphatase activity of 0.648 (U/mL). Based on the quadratic model obtained from the Central Composite Design, the maximum predicted enzymatic activity reached approximately 2.79 (U/mL), corresponding to a 3.3-fold increase compared to preliminary conditions. However, experimental validation under optimized conditions resulted in an activity of 1.96 ± 0.14 (U/mL), confirming the reliability of the model and demonstrating a substantial improvement in enzyme production.

*T. asperellum* showed intermediate results when compared to the two microorganisms above. In a supplemented culture medium, with sucrose in the concentration range of 11 to 17.5 (g/L), yeast extract from 6 to 10.5 (g/L), and KH_2_PO_4_ from 0 to 0.2 (g/L), it had a maximum enzymatic activity of 0.650 (U/mL), while before the average activity was around 0.405 (U/mL), an increase of approximately 60% from the initial value.

### Validation of culture media suggested by CCD for each microorganism studied

In this stage, the reproducibility of the results of the models proposed by the CCD was verified when the experimental conditions indicated by the analysis of response surfaces and contour curves were employed. Validation was performed in triplicate for each selected culture medium per microorganism.

**Fig. 15:**
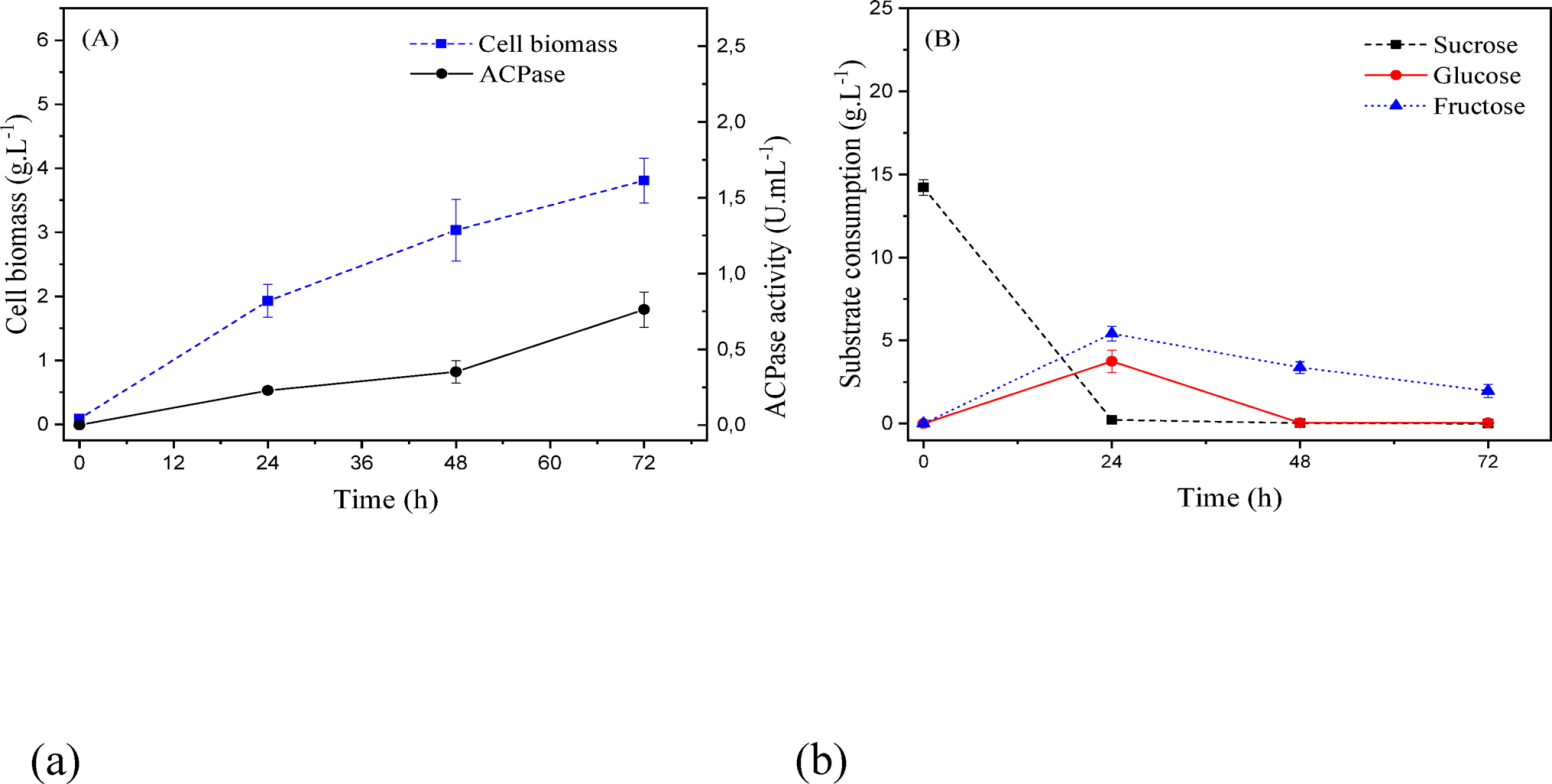
(a) Cellular biomass (g/L) and acid phosphatase activity (U/mL); (b) substrate consumption (sucrose, glucose, and fructose) during submerged fermentation of *T. asperellum* under optimized conditions defined by the Central Composite Design at 25 °C and 150 rpm

The culture medium for *T. asperellum*, which maximized acid phosphatase activity, was within the following concentration ranges (g/L): [11.0–17.5] sucrose, [6–10.5] yeast extract, and [0–0.2] KH_2_PO_4_. It was decided to work with a culture medium composed of 15.0 (g/L) sucrose, 8.0 (g/L) yeast extract, and 0.2 (g/L) monobasic potassium phosphate, a culture medium titled MSO-Ta. Figure 15 (a) shows the kinetics of cellular growth and enzymatic activity, reaching an average final value of 3.81 ± 0.35 (g/L) and 0.76 ± 0.12 (U/mL), respectively. Similar improvements in enzyme production after process optimization have been reported in recent studies using low-cost substrates and controlled fermentation strategies (Wang et al. 2024).

The carbohydrate used in the culture media was sucrose, which is hydrolyzed into glucose and fructose. Analyzing Fig. 15 (b), this microorganism consumes all sucrose within 24 h, with one part used for its cellular growth, and the other transformed into glucose and fructose. Glucose, being an easily absorbed carbohydrate, is completely consumed within 48 h, according to the kinetics. The same does not occur for fructose, leaving a residue of approximately 2.0 (g/L) in the culture medium.

According to the CCD, the fungus *T. harzianum* had an intermediate enzyme production potential when compared to the other microorganisms. The highest acid phosphatase activities produced by this microorganism were for the concentration ranges (g/L): [14.5–22.0] sucrose, [3.5–7.0] yeast extract, and [0.6–1.1] monobasic potassium phosphate. Therefore, the selected medium was composed of 18.0 (g/L) sucrose, 5.0 (g/L) yeast extract, and 0.8 (g/L) monobasic potassium phosphate, a culture medium titled MSO-Th.

**Fig. 16:**
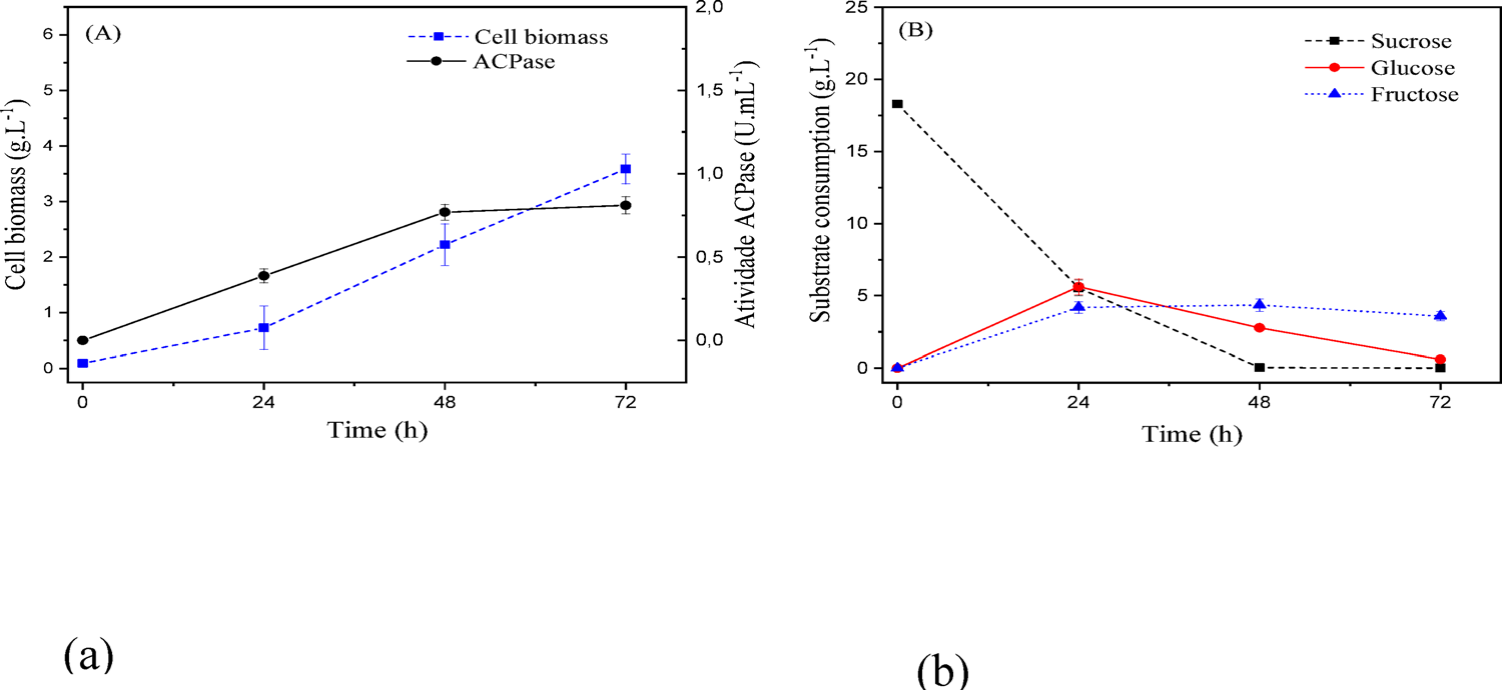
(a) Cellular biomass (g/L) and acid phosphatase activity (U/mL); (b) substrate consumption (sucrose, glucose, and fructose) during submerged fermentation of *T. harzianum* under optimized conditions defined by the Central Composite Design at 25 °C and 150 rpm

Figure 16 (a) shows the kinetics of cellular growth and enzymatic activity, reaching an average final value of 3.58 ± 0.27 (g/L) and 0.81 ± 0.05(U/mL), respectively.

As this microorganism showed the lowest cellular growth compared to the others, substrate consumption was lower, leaving a higher residual concentration in the medium. Compared to *T. asperellum*, its carbohydrate consumption was slower, requiring twice the time to consume sucrose, i.e., 48 h. This result also affected glucose and fructose consumption. The former was almost completely consumed after 72 h, and the latter was partially consumed, leaving a residue of approximately 3.5 (g/L) in the culture medium.

The best results were for *T. reesei*. The culture medium that maximizes enzymatic activity was within the concentration ranges (g/L): [20.0–26.0] sucrose, [6.0–9.5] yeast extract, and [1.6–2.0] monobasic potassium phosphate. It was decided to work with the medium composed of 23.4 (g/L) sucrose, 8.0 (g/L) yeast extract, and 1.84 (g/L) KH_2_PO_4_, a culture medium titled MSO-Tr.

**Fig. 17:**
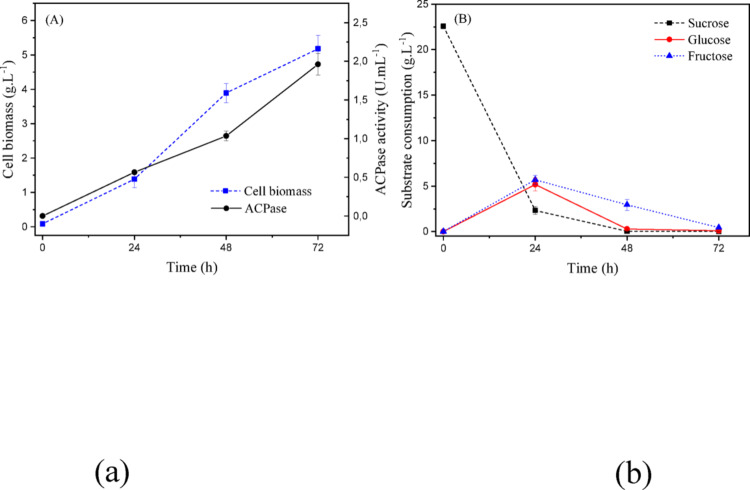
(a) Cellular biomass (g/L) and acid phosphatase activity (U/mL); (b) substrate consumption (sucrose, glucose, and fructose) during submerged fermentation of *T. reesei* under optimized conditions defined by the Central Composite Design at 25 °C and 150 rpm

The results are presented in Fig. 17, the highlight of cellular biomass growth and enzymatic activity is notable, reaching an average final value of 5.32 ± 0.48 (g/L) and 1.96 ± 0.14 (U/mL), respectively. Sucrose and glucose consumption was complete after 48 h of fermentation, while fructose recorded minimal residues of approximately 0.5 (g/L) at the end of the process.

**Fig. 18:**
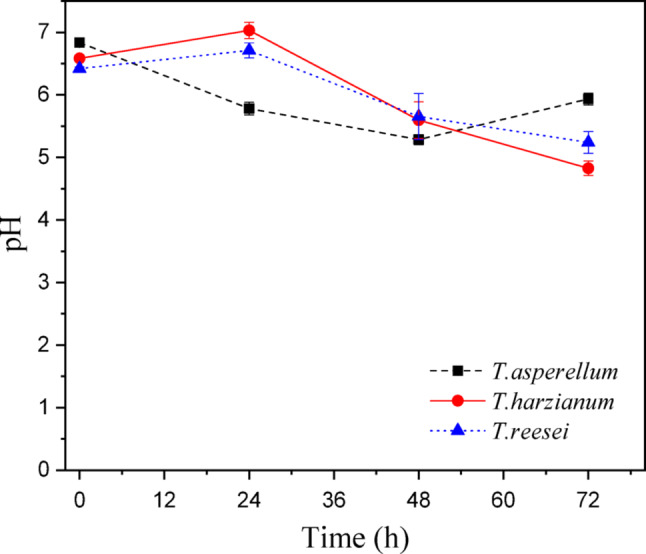
pH variation during 72 h of submerged fermentation of *Trichoderma spp.* cultivated in optimized media obtained by Central Composite Design at 25 °C and 150 rpm. Data represent mean values of triplicate experiments

Figure 18 shows the average pH variation for the three microorganisms. The initial pH ranged from 6.5 to 6.8, given that the culture media for each *Trichoderma* strain had different concentrations and no initial correction was made, following the same CCD procedures.

In general, the pH behavior was satisfactory, as all remained in the acidic range during this batch time (Fig. 18). The culture media fermented by *T. harzianum* and *T. reesei* showed similar behaviors, remaining practically constant in the first 24 h, and reaching values close to 5.0 at the end of the process. Several authors report that the optimal pH for acid phosphatase activity varies between 3.5 and 6.0, depending on the producing microorganism (García et al. 2004; Rombola et al. 2014). Most soil microorganisms from the Brazilian savanna can produce enzymes of the acid phosphatase class, since the pH of these soils varies between 4.9 and 5.7 (Purcena et al. 2014).

Souza et al. (2016), starting from an initial concentration of 107 (spores/mL) of *T. harzianum* in culture medium supplemented with 15 (g/L) glucose and without the addition of inorganic phosphate, obtained 14.3 (U/mg) as maximum specific enzyme activity in 48 h of fermentation. After this time, they observed a reduction in this activity and believe it was due to the formation of inorganic phosphate present in the culture medium. The working pH was 4.0, being controlled throughout the 90 h of the process.

Some works involving acid phosphatase production and cellular growth of *T. harzianum* ALL42 indicate inhibition and protein secretion at high glucose concentrations. Ramada et al. (2015) detected low total protein production for this species when grown in 2.0% glucose medium. Lima (2006) observed that *T. harzianum* ALL 42, when inoculated in a medium with only glucose as a carbon source, secreted less protein compared to productions carried out in medium supplemented with corn, soybean meal, commercial starch, and animal feed.

Acid phosphatase production by this microorganism was proportional to the amount of glucose added in Souza’s (2011) work. The fungus *T. harzianum* ALL42, when grown in selective medium with 1.5% glucose, showed an activity of 0.17 (U/mL), being 21% higher than the activity observed in the same medium, but with 0.5% of this carbohydrate.

### SDS-PAGE results

**Fig. 19:**
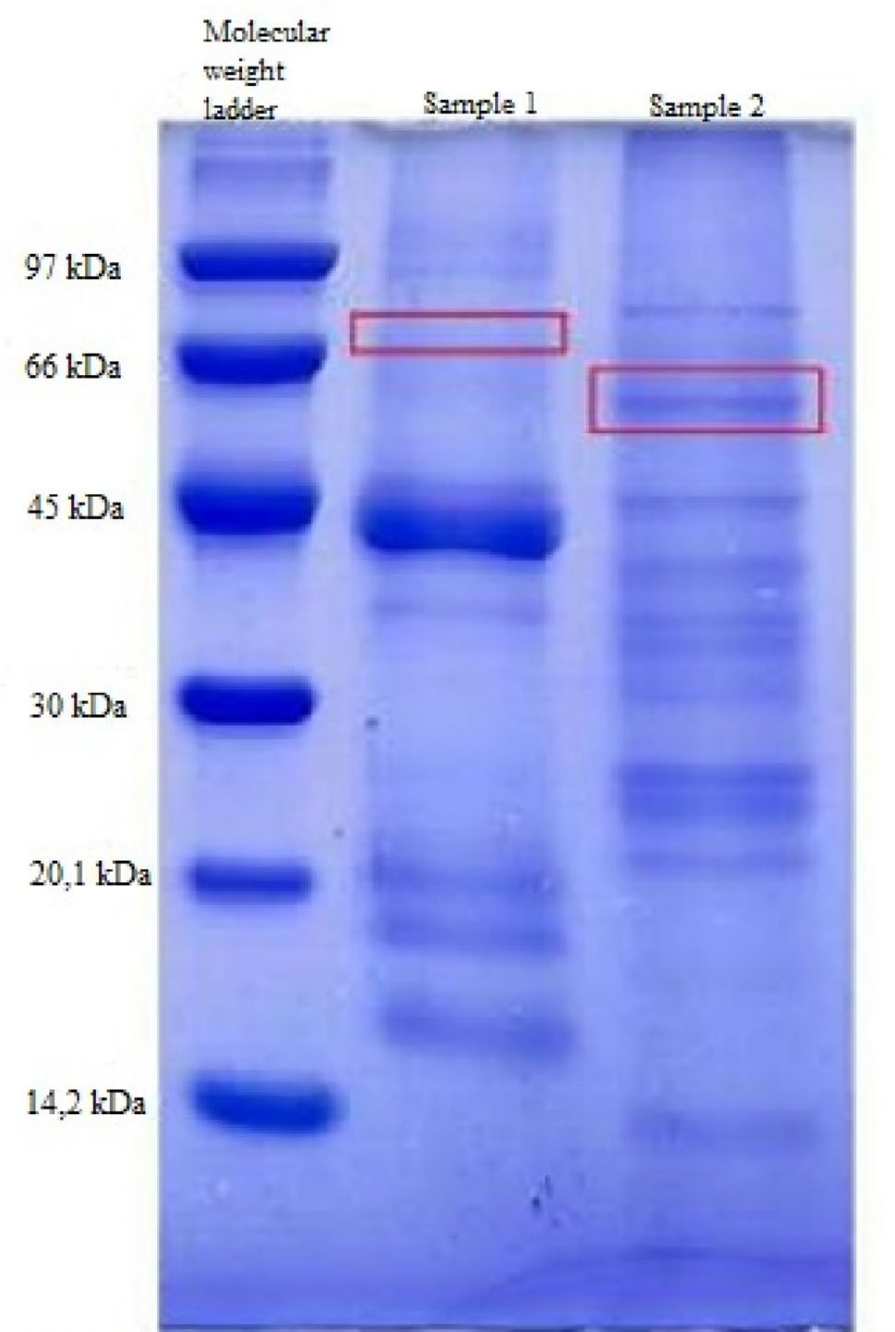
SDS-PAGE (12%) analysis of extracellular protein extracts. Lane 1: molecular weight marker (10–250 kDa); Lane 2: commercial acid phosphatase from potato (~ 69 kDa); Lane 3: commercial acid phosphatase from wheat germ (~ 58 kDa). Protein bands observed in the *Trichoderma spp.* extracts are within the expected molecular weight range for fungal acid phosphatases

SDS-PAGE analysis of the extracellular protein extracts revealed multiple protein bands, indicating the presence of a complex mixture of secreted proteins (Fig. 19). Due to the use of crude extracts and the absence of enzyme purification or activity staining, it was not possible to unambiguously assign a specific protein band to acid phosphatase in the *Trichoderma* samples.

Commercial acid phosphatases from wheat germ and potato, analyzed under the same conditions, exhibited prominent bands at approximately 58 and 69 kDa, respectively. Fungal acid phosphatases reported in the literature, including those from *Trichoderma* species, typically present molecular weights within this range, depending on the species and post-translational modifications. Therefore, while bands observed in the *Trichoderma* extracts are compatible with the expected molecular weight range of acid phosphatases, no definitive assignment is claimed in this study.

##  Conclusions

This study demonstrated the potential of *Trichoderma spp.* as efficient producers of acid phosphatase under submerged fermentation using low-cost culture media. The statistical optimization approach enabled the identification of key cultivation parameters that significantly influenced enzyme production, contributing to improved yields while maintaining process simplicity and economic feasibility.

Beyond production optimization, complementary protein profiling supported the characterization of the extracellular enzyme extracts. SDS-PAGE analysis revealed protein bands within the molecular weight range typically reported for fungal acid phosphatases, confirming the successful production of these enzymes under the optimized conditions.

Taken together, the results highlight the effectiveness of the optimized fermentation strategy and the biotechnological relevance of the acid phosphatases obtained. This work contributes to the development of sustainable and cost-effective approaches for acid phosphatase production and reinforces the applicability of *Trichoderma*-derived enzymes in industrial and biotechnological contexts.
